# 4-Hydroxyphenylpyruvate Dioxygenase-Like Protein Promotes Pancreatic Cancer Cell Progression and Is Associated With Glutamine-Mediated Redox Balance

**DOI:** 10.3389/fonc.2020.617190

**Published:** 2021-01-18

**Authors:** Xianglai Ye, Xiujuan Wei, Jing Liao, Peipei Chen, Xueyun Li, Yulong Chen, Yue Yang, Qiongya Zhao, Hongwei Sun, Liming Pan, Guorong Chen, Xujun He, Jianxin Lyu, Hezhi Fang

**Affiliations:** ^1^ Key Laboratory of Laboratory Medicine, Ministry of Education, Wenzhou Medical University, Wenzhou, China; ^2^ Zhejiang Provincial Key Laboratory of Medical Genetics, Department of Cell Biology and Medical Genetics, Wenzhou Medical University, Wenzhou, China; ^3^ College of Laboratory Medicine and Life Sciences, Wenzhou Medical University, Wenzhou, China; ^4^ College of Laboratory Medicine, Hangzhou Medical College, Hangzhou, China; ^5^ Department of Surgery, The First Affiliated Hospital of Wenzhou Medical University, Wenzhou, China; ^6^ Department of Pathology, The People’s Hospital of Yuhuan, The Yuhuan Branch of the First Affiliated Hospital of Wenzhou Medical University, Taizhou, China; ^7^ Department of Pathology, The First Affiliated Hospital of Wenzhou Medical University, Wenzhou, China; ^8^ Department of Laboratory Medicine, Zhejiang Provincial People’s Hospital, Affiliated People’s Hospital of Hangzhou Medical College, Hangzhou, China

**Keywords:** mitochondria, pancreatic ductal adenocarcinoma, metabolic reprogramming, glutamine, redox balance

## Abstract

Tumor cells develop a series of metabolic reprogramming mechanisms to meet the metabolic needs for tumor progression. As metabolic hubs in cells, mitochondria play a significant role in this process, including energy production, biosynthesis, and redox hemostasis. In this study, we show that 4-hydroxyphenylpyruvate dioxygenase-like protein (HPDL), a previously uncharacterized protein, is positively associated with the development of pancreatic ductal adenocarcinoma (PDAC) and disease prognosis. We found that overexpression of HPDL in PDAC cells promotes tumorigenesis *in vitro*, whereas knockdown of HPDL inhibits cell proliferation and colony formation. Mechanistically, we found that HPDL is a mitochondrial intermembrane space localized protein that positively regulates mitochondrial bioenergetic processes and adenosine triphosphate (ATP) generation in a glutamine dependent manner. Our results further reveal that HPDL protects cells from oxidative stress by reprogramming the metabolic profile of PDAC cells toward glutamine metabolism. In short, we conclude that HPDL promotes PDAC likely through its effects on glutamine metabolism and redox balance.

## Introduction

Of all the cancer types, pancreatic cancers have the lowest 5-year survival rate at 9% (1). Pancreatic ductal adenocarcinoma (PDAC), as the most common type of pancreatic cancer, is a lethal cancer type as it is typically diagnosed late resulting in poor prognosis (2, 3). Increased biosynthetic demand for proliferation drives tumor cells to reprogram cellular metabolism (4). In PDAC, composed of a minority of malignant ductal cells with a dearth of blood vessels and an existence of extensive desmoplastic stroma originating from cancer-associated fibroblasts (CAFs), tumor cells live in a microenvironment of poor perfusion, poor nutrient, hypoxia and acidosis (5–7). To survive in this environment, PDAC tumor cells develop a series of metabolic reprograming mechanisms involving aerobic glycolysis, glutaminolysis, fatty acid oxidation, and protein scavenging to fuel the energy demand and biosynthetic process essential for cell growth (8–10). The oncogene *KRAS* and tumor suppressor genes *TP53*, *CDKN2A*, and *SMAD4* play a significant role in pancreatic neoplasia (11). As PDAC progresses to malignancy, called acinar-to-ductal metaplasia (ADM), oncogenic mutations of *KRAS* essentially initiate precursor lesions called pancreatic intraepithelial neoplasias (PanINs) (12).

The role of mitochondria is not typically considered important in tumor cells because they predominantly utilize glucose to generate ATP *via* aerobic glycolysis rather than mitochondrial oxidative phosphorylation (OXPHOS) (13). Since the mitochondria is the central organelle in cell metabolism where most bioenergetic and biosynthetic processes take place, they play a pivotal role in driving metabolic reprogramming (14, 15). oncogenes and tumor suppressor genes tightly regulate mitochondrial metabolism to meet metabolic need in the neoplasia process (16). Glutamine is a non-essential amino acid that can be used as a substrate in the tricarboxylic acid (TCA) cycle in mitochondria. Glutaminase (GLS), which converts glutamine to glutamate in the TCA cycle, is upregulated by the oncogene MYC-mediated mTOR pathway (17). Moreover, oncogenic mutation of *KRAS* reprograms glutamine metabolism by upregulating aspartate transaminase (GOT1) and downregulating glutamate dehydrogenase (GLUD1) at the transcription level (18).

We previously showed that the mitochondrial proteins COX6B2 and HSP60 play significant roles in the promotion of PDAC (19, 20). In addition, a recent study revealed that overexpression of the mitochondrial protein UQCRC1 from the electron transfer chain (ETC) promotes PDAC growth by enhancing OXPHOS and ATP generation (21). Since reactive oxygen species (ROS) production occurs in the ETC, redox balancing is required in all cancer cells with glutamine responsible for generating antioxidant NADPH and glutathione (GSH) (22). To account for this, a mitochondrion-localized protein SLC1A5 variant, functions as a glutamine transporter in the mitochondria, and mediates redox balancing in mitochondria (23). More and more studies have shown a close relation between PDAC and mitochondrion-localized proteins, and suggest that the role of mitochondrial proteins in the progression of PDAC cannot be ignored.

**Figure 1:**
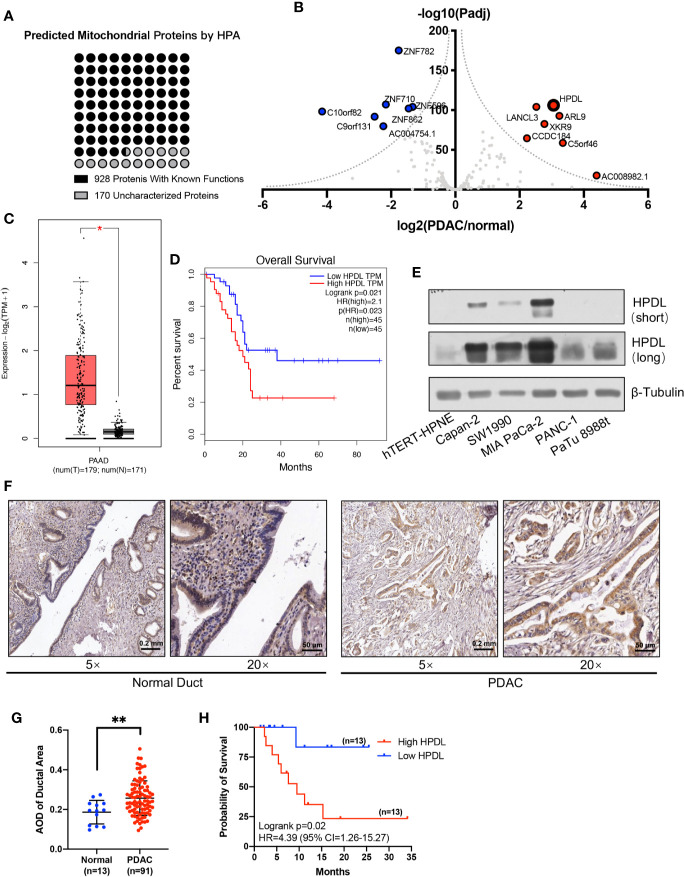
HPDL is upregulated in pancreatic ductal adenocarcinoma and associated with poor prognosis. **(A)** Mitochondrial proteins that experimentally detected in the mitochondria by the Human Protein Atlas (http://www.proteinatlas.org). The functional definitions of these mitochondrial proteins were based on the annotation of Uniprot (https://www.uniprot.org). **(B)** Gene expression profile of function-unknown mitochondrial proteins based on the data generated by the TCGA (https://www.cancer.gov/tcga) and GTEx (https://commonfund.nih.gov/GTEx) datasets. The genes were plotted with expression of PDAC/normal duct and adjusted p-value. The red points refer to genes up-regulated in PDAC and the blue points refer to genes down-regulated in PDAC. **(C, D)** Expression plot and survival plot of HPDL in pancreatic adenocarcinoma (PAAD) made by GEPIA server (http://gepia.cancer-pku.cn). For expression plot **(C)**, threshold was set as follows: log_2_FoldChange <1, p-value <0.01. For survival plot **(D)**, high and low expression groups were split by quartile, and the hazards ratio (HR) was calculated based on Cox PH model. **(E)** Western blot analysis of HPDL in different human pancreatic adenocarcinoma cell lines and human immortalized pancreatic duct cell line hTERT-HPNE. β-Tubulin was used as loading control. **(F)** Immunohistochemical (IHC) analysis of tissue microarray (TMA). TMA was incubated with anti-HPDL and stained with DAB and hematoxylin. HPDL expression in normal and malignant pancreatic ducts was shown in different zoom levels. **(G, H)** Quantitative analysis of HPDL expression in TMA. Quantification of HPDL expression was represented as average optical density (AOD). Comparisons between normal and malignant pancreatic ducts **(G)** was performed. Survival curve **(H)** was performed between low HPDL group and high HPDL group which were split by median. (*P < 0.05. **P < 0.01.)

According to the subcellular map of the Human Protein Atlas (HPA), mitochondrial proteins account for 6% of all proteins and play an essential part in various cellular processes (24). There are abundant publications and experimental evidence for the function of mitochondrial proteins, but there are still 170 proteins whose functions remain to be defined **(** **Figure 1A** **)**. In this study, we describe a mitochondrion-localized protein, 4-hydroxyphenylpyruvate dioxygenase like protein (HPDL). The function of HPDL has not been previously defined, apart from its association with neurodegenerative diseases (25). For this study we performed a series of experiments to test the effects of HPDL, including its effect on PDAC progression, mitochondrial function, and cellular metabolism.

## Materials and Methods

### Cell Lines and Cell Culturing

Human pancreatic cell lines, PaTu 8988t, MIA PaCa-2, PANC-1, SW1990, Capan-2, and the immortalized pancreatic ductal cell line hTERT-HPNE, were obtained from the Cell Bank of the Chinese Academy of Science (Shanghai, China). Cells were cultured in Dulbecco’s modified Eagle’s medium (DMEM; Sigma-Aldrich, St. Louis, MO, USA) containing 4.5 g/L glucose, 4 mM glutamine, 110 mg/L sodium pyruvate, supplemented with 10% fetal bovine serum (FBS, Sigma-Aldrich), 100 U/ml penicillin (Beyotime, Shang, China), 0.1 mg/ml streptomycin (Beyotime), and 0.25 μg/ml amphotericin B (MP Biomedicals, Santa Ana, CA, USA). Cells were cultured in a CO2 incubator (Thermo Fisher Scientific, Waltham, MA, USA) at 37°C under 5% CO2.

### Generation of Stable Knockdown and Overexpression Cell Lines

Stable knockdown (KD) and overexpression (OE) models were generated based on a previous protocol using second-generation lentiviruses (26). Lentiviral particles were generated by co-transfection of 5×10^5^ HEK293T cells (Cell Bank of Chinese Academy of Science) in a 6-well plate with packaging vectors psPAX2 (1 μg), envelope vectors pMD2.G (0.5 μg), and shRNA sequence containing knockdown vectors pLVSHG01 (1 μg) or HPDL protein coding sequence NM_032756.3 containing overexpression vectors pLVEXP01 (1 μg) (Cyagen, Santa Clara, CA, USA). Transfection was performed using Lipofectamine 3000 (Thermo Fisher Scientific). The HPDL shRNA and scramble shRNA sequences are shown as follows: 5’-AGTCCGTACAGGAGCAATCTG-3’ (HPDL), 5’-CCTAAGGTTAAGTCGCCCTCG-3’ (scramble). Monoclonal selection of stable cell lines was performed based on a previously described limiting dilution method (27).

### Immunohistochemical Analysis of Tissue Microarray

Immunohistochemical (IHC) analysis of tissue microarray (TMA) was performed as previously described (28). Ninety-one malignant tumor pancreatic tissue sample and 13 non-cancerous tumor pancreatic tissue samples were obtained from Zhejiang Provincial People’s Hospital. Experimental procedures were approved by Ethical Committee of Wenzhou Medical University (grant number: 2019-084). In short, paraffin-embedded tissues were punched into the recipient block to make the TMA, and the TMA section were then placed on a slide. After deparaffinization and hydration, endogenous peroxidase was blocked with 0.3% hydrogen peroxide (H_2_O_2_) and heat-induced epitope retrieval (HIER) was performed. Finally, the TMA was incubated with anti-HPDL (1:250) and stained with diaminobenzidine (DAB) and hematoxylin.

### Immunofluorescence Staining

Cells in φ14mm slides were fixed with 4% paraformaldehyde and permeabilized with 0.1 mM digitonin (Sigma-Aldrich). Then, permeabilized cells were blocked with 5% goat serum, followed by primary antibodies and fluorescent-conjugated secondary antibodies. Primary and secondary antibodies was shown in **Supplementary Table 1**. The nucleus was stained by DNA binding stain 4,6-diamidino-2-phenylindole (DAPI). The fluorescence in cells was detected using an A1 confocal laser microscope (Nikon, Minato-ku, Tokyo, Japan).

### SDS PAGE, Blue Native PAGE, and Immunoblotting

For SDS PAGE, cells were lysed in RIPA buffer (Cell Signaling Technology, Danvers, MA, USA) supplemented with protease inhibitor phenylmethylsulfonyl fluoride (PMSF, 1mM, Sangon Biotech, Shanghai, China), and heated to 95°C for 5 min. Blue native PAGE was performed according to a previously described protocol (29). For blue native PAGE, cells were lysed in solubilization buffer (50 mM NaCl, 50mM imidazole, 2 mM 6-aminohexonic acid, 1mM EDTA, pH 7.00) and supplemented with 2.5% digitonin (w/v, Sigma-Aldrich) and 1mM PMSF (Sangon Biotech). Proteins resolved by SDS-PAGE or blue native PAGE were transferred to a PVDF membrane (Bio-Rad, Hercules, CA, USA). Membranes were then immunoblotted with primary antibodies and secondary antibodies. Primary and secondary antibodies was shown in **Supplementary Table 1**.

### Cell Proliferation, Colony Formation, and Wound Healing Assay

Cell proliferation was analyzed by counting cells in a 12-well plate with a NovoCyte flow cytometer (Agilent, Santa Clara, CA, USA). Colony formation was analyzed by seeding 1,000 cells in a 6-well plate for 10 days, and cells were fixed with 4% paraformaldehyde and stained with crystal violet (Beyotime). The size of the clones was measured using ImageJ (30). The wound healing assay was performed as previously described (31). 1×10^6^ cells were seeded in a 6-well plate and the gaps were created by scratching on the monolayer of cells with a pipette tip. The gap size was measured using the Wound Healing Coherency Tool in ImageJ.

### Isolation of Mitochondria

Mitochondrial isolation was performed as previously described (32). Cells was suspended in a hypotonic isolation buffer (0.1×IB, 3.5 mM Tris-HCl, 2.5 mM NaCl, 0.5 mM MgCl_2_, pH 7.80) on ice to break the cell membrane integrity and then homogenized 20-30 strokes with a Dounce homogenizer (WHEATON, Millville, NJ, USA). The homogenate was then incorporated with 1/10 volume of hypertonic buffer (10×IB, 350 mM Tris-HCl, 250 mM NaCl, 50 mM MgCl_2_, pH 7.80) to restore the isotonic solution. Centrifugation was performed to eliminate the contamination from nuclei, unbroken cells, and other debris. Multiple centrifugations under a low speed (1,200 × g × 3 min) were performed to remove the waste in the pellets. Finally, the mitochondria were pelleted down at 15,000 × g for 2 min and washed with homogenization buffer A (10mM Tris-HCl, 320mM sucrose, pH 7.40) to yield crude mitochondria.

### Cell Component Separation and Mitochondrial Sub-Fractionation Analysis

The cell components were separated using a Nuclear and Cytoplasmic Protein Extraction Kit (Beyotime) according to the manufacturer’s instructions. After treatment with hypotonic extraction buffer, cells were fully swollen, and cytoplasmic proteins were released. Nuclear pellets were obtained by centrifugation at 16,000 × g for 10 min. Nuclear extraction buffer was added to nuclei and a vortex was used several times to obtain the nuclear protein.

For mitochondrial sub-fractionation analysis, two different methods were performed as previously described (33, 34). To separate the soluble components (mitochondrial intermembrane space and matrix) and insoluble components (mitochondrial outer membrane and inner membrane), isolated mitochondria were treated with 0.1 M Na_2_CO_3_ on ice for 30 min, followed by ultracentrifugation (Beckman Coulter, Brea, CA, USA) at 100,000 g for 45 min. To further separate the mitochondrial components, mitochondria were treated with hypotonic buffer (10 mM HEPES/KOH, pH 7.40) on ice for 20 min to break the outer membrane. Broken mitochondria were deposited at 1,900 g for 15 min, and the supernatant was subsequently centrifuged at 50,000 × g for 30 min to separate the proteins in the outer membrane and the intermembrane space.

### Oxygen Consumption Rate Assay

OCR was measured using Oxygraph-2k (OroborOSX, Innsbruck, Austria) according to the manufacturer’s instructions (35). Cells (1×10^6^) were added to the chamber, and the basal respiration of cells was measured. ATP synthase inhibitor oligomycin (2.5 μM, Sigma-Aldrich) and uncoupler carbonyl cyanide 4-(trifluoromethoxy) phenylhydrazone (FCCP, 5 μM, Sigma-Aldrich) were added to measure the non-phosphorylating proton leak and maximum non-coupled flux, respectively.

### Adenosine Triphosphate Assay and Hydrogen Peroxide Assay

ATP determination was performed using an ATP Bioluminescent Assay Kit (Sigma-Aldrich) according to the manufacturer’s instructions. Intracellular ATP was released by treating 5×10^5^ cells with ATP-releasing reagent. Free ATP was detected by light produced through a luciferin-based reaction.

H_2_O_2_ determination was performed using a Hydrogen Peroxide Assay Kit (Beyotime) according to the manufacturer’s instructions. Cells (1×10^6^) were sonicated in H_2_O_2_ extraction buffer and intracellular H_2_O_2_ oxidized Fe^2+^ in xylenol orange solution to generate color. The absorbance was measured at 560 nm.

### Mitochondrial DNA Copy Number Analysis

Quantitative PCR was performed using SYBR Green Supermix (Bio-Rad) with Quantagene q225 (Kubo Tech, Beijing, China). Primers for mtDNA and nuclear DNA-encoded ACTIN are shown as follows. F: 5’-CACCCAAGAACAGGGTTTGT-3’, R: 5’-TGGCCATGGGTATGTTGTTAA-3’ (mtDNA). F: 5’-AGCTGTCACTCCAGGGTCC-3’, R:5’-GATCCACATCTGCTGGAAGGT-3’ (ACTIN).

### Citrate Synthase Assay

Citrate synthase activity was determined using a previously described protocol (36). Acetyl-CoA (0.3 mM, Sigma-Aldrich) and oxaloacetic acid (0.5 mM, Sigma-Aldrich) were used as substrates for citrate synthase and 5,5-dithiobis (2-nitrobenzoic acid) (DTNB, 0.1mM, Sigma-Aldrich) were used to generate color. The reaction was activated with 10 μg snap-freeze cell lysate in reaction buffer (0.1 M Tris-HCl, pH 8.00). The absorbance was measured at 412 nm using a SpectraMax iD3 Multi-Mode Microplate Reader (Molecular Devices, Shanghai, China).

### Mitochondrial Morphology Analysis

Mitochondrial morphology was analyzed by immunofluorescence staining and transmission electron microscopy (TEM). For immunofluorescence staining, analysis was performed as stated above, with HSP60 used as a mitochondrial indicator. Mitochondrial morphology was analyzed using a macro tool MINA (37) in the ImageJ software (30).

For TEM, 5×10^6^ cells were fixed with 2.5% glutaraldehyde and 1% osmic acid at 4°C and stained with uranyl acetate for 1 h. Gradient dehydration was performed in 30%, 50%, 70%, 80%, 90%, and 100% acetone for 15 min each. Then, a resin-based gradient embedding was performed at 37°C followed by 65°C overnight. Finally, sectioned slides were observed under a H-7500 electron microscope (Hitachi, Chiyoda-ku, Tokyo, Japan).

### Transcriptome Sequencing

RNA library construction was performed using a NEBNext Ultra RNA Library Prep Kit (New England Biolabs, Ipswich, MA, USA). mRNA was enriched with oligo(dT) beads and fragmented in fragmentation buffer. Then, reverse transcription was done using the M-MuLV system with random hexamer primer. cDNA fragments (200–250 bp) were purified with AMpure XP beads (Beckman Coulter, Beverly, CA, USA). Clustering and sequencing were performed using a TruSeq PE Cluster Kit and NovaSeq platform (Illumina, San Diego, CA, USA).

### Liquid Chromatography-Mass Spectrometry Analysis

Quasi-targeted metabolic profiling analysis of cells was performed on a QTRAP6500+ mass spectrometer coupled with an ExionLC AD system (all from SCEIX, Framingham, MA, USA). Cells (1×10^7^) were sonicated in 80% methanol and the centrifuged supernatant was subsequently injected into LC/MS. For the positive ion mode, samples were injected into a BEH C8 column (100×2.1 mm, 1.9 μm), and for the negative ion mode, samples were injected into an HSS T3 column (100×2.1 mm, 1.8 μm). A 25-min gradient containing flow phase A (0.1% formic acid-water) and flow phase B (0.1% formic acid-acetonitrile) was performed at a flow rate of 0.35 ml/min. For MS, the IonSpary Voltage was set at 5500V and -4500V, respectively. Metabolites were identified using multiple reaction monitoring (MRM) based on the Novogene self-built database (Novogene, Beijing, China).

Targeted metabolite analysis was performed on a Xevo G2-XS QTof mass spectrometry coupled with an ACQUITY UPLC system, and data analysis was done with Progenesis QI (all from Waters, Milford, MA, USA). Metabolites in a homogenate of 1×10^6^ cells were extracted using chloroform, methanol, and water at a ratio of 8:4:3 and resuspended in 1% acetonitrile. For UPLC, samples were injected on an HSS T3 column (100×2.1 mm, 1.8 μm) using an 8-min gradient containing flow phase A (0.1% formic acid-water) and flow phase B (0.1% formic acid-acetonitrile) at a flow rate of 0.5 ml/min. For the negative ion mode, the capillary energy and sample cone were set as 2000V and 20V, respectively. Scan time was set at 0.1 s intervals for 60 s.

### Bioinformatic Analysis and Statistical Analysis

Gene expression analysis and survival analysis from TCGA and GTEx databases were performed using the GEPIA server (38). The mitochondrial targeting sequence was predicted by the MitoProt server (39). Bioinformatic analysis was performed using R package. The enrichment of differentially expressed genes or metabolites was based on the KEGG pathways (40). All quantitative experiments were performed using a minimum of three independent replicates. The quantitative results are presented as mean ± SD, and the p value was calculated using a student’ s *t* test with SPSS v22.0 (IBM, Chicago, IL, USA). Statistical graphs were made using Prism 8 (GraphPad Software, San Diego, CA, USA).

## Results

### HPDL Is Upregulated in Pancreatic Ductal Adenocarcinoma and Is Associated With Poor Prognosis

We profiled gene expression in PDAC using data from TCGA and GTEx databases **(** **Figure 1B** **)**. Our results found that *HPDL* (HGNC:28242) was significantly upregulated, with considerable fold change, in PDAC. *HPDL*, also known as *GLOXD1* (Glyoxalase Domain-Containing Protein 1), is a paralog of *HPD* (4-Hydroxyphenylpyruvate Dioxygenase). It is a protein coding gene for 4-hydroxyphenylpyruvate dioxygenase like protein. Until now, the function of HPDL has been unclear.

We plotted *HPDL* expression and overall survival rate (OS) in PDAC and found upregulation was associated with poor prognosis in PDAC **(** **Figures 1C, D** **)**. In pancreatic tumor cell lines, HPDL was also detected at the protein level, especially in MIA PaCa-2, compared with immortalized normal pancreatic duct cell line hTERT-HPNE **(** **Figure 1E** **)**. We also performed immunohistochemistry (IHC) staining of 91 malignant tumor pancreatic tissue samples and 13 non-cancerous tumor pancreatic tissue samples. We quantified the staining intensity in selected ductal areas and found that the protein level of HPDL was upregulated in pancreatic malignant tumors **(** **Figures 1F, G** **)**. We followed up on 26 cases and split them into two groups by median HPDL staining intensity. The survival plot showed that the high HPDL group had a lower overall survival rate **(** **Figure 1H** **)**. Collectively, HPDL is upregulated in PDAC at both the mRNA and protein levels, in both tumor cell lines and tumor tissues, and high expression of HPDL is associated with poor prognosis in PDAC patients.

### HPDL Promotes Pancreatic Ductal Adenocarcinoma *In Vitro*

**Figure 2:**
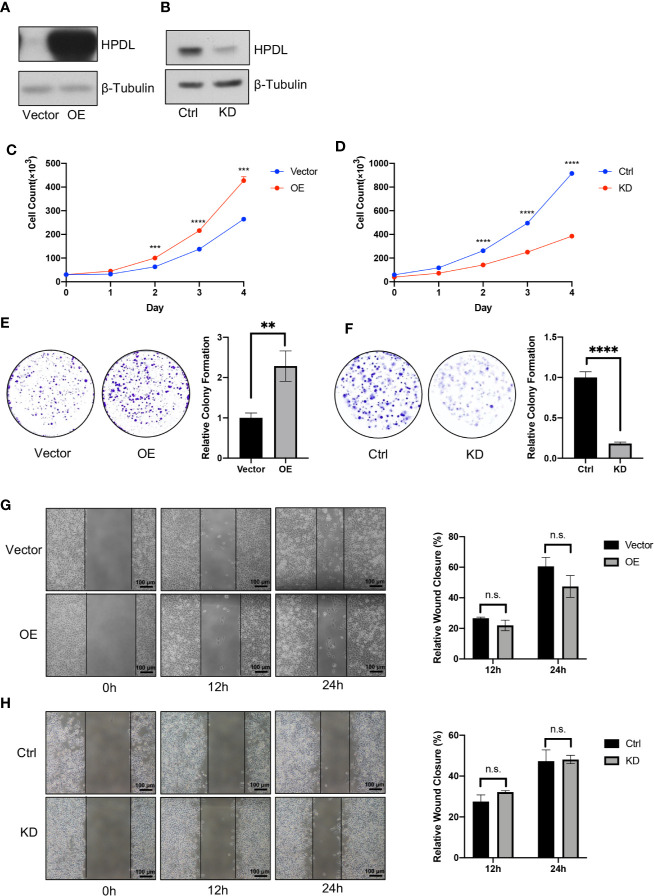
HPDL promotes pancreatic ductal adenocarcinoma *in vitro*. **(A, B)** Validation of HPDL overexpression **(A)** and knockdown **(B)** cells with theirs paired control cells. HPDL overexpression (OE) model and its empty vector control (Vector) were generated in PaTu 8988t, HPDL knockdown (KD) model and its scramble control (Ctrl) were generated in MIA PaCa-2. Cell lysate was separated with SDS-PAGE and immunoblotted with antibodies as indicated. β-Tubulin was used as loading control. **(C, D)** Cell proliferation of HPDL OE **(C)** and KD **(D)** cells with theirs paired control cells. Cells were seeded in 12-well plate and counted for 4 days. **(E, F)** Colony formation analysis of HPDL OE **(E)** and KD **(F)** cells with theirs paired control cells. Cells were seeded in 6-well plate and stained with crystal violet after 10-day culture. **(G, H)** Wound healing analysis of HPDL OE **(G)** and KD **(H)** cells with theirs paired control cells. Cells were seeded in 6-well plate and scratched with a pipette tip. Wound closure was measured at 0, 12, and 24 h. (**P < 0.01. ***P < 0.001. ****P<0.0001. n.s., no significance.)

To further investigate HPDL in PDAC, we generated two cell models in pancreatic tumor cell lines **(** **Figures 2A, B** **)**. The overexpression (OE) model was generated in PaTu 8988t due to its relatively low expression among other pancreatic tumor cell lines **(** **Figure 1E** **)**, which may imitate the functional effect of HPDL. For the same reason, a knockdown (KD) model was generated in the MIA PaCa-2 line which demonstrated high HPDL expression. In accordance with our bioinformatical analysis and IHC data, HPDL OE cells demonstrated better proliferation and HPDL KD cells had worse proliferation compared to control cells **(** **Figures 2C, D** **)**. In colony formation assays, HPDL OE and KD cells followed the same pattern **(** **Figures 2E, F** **)**.

HPDL was observed to promote PDAC growth in these two experiments, and we next tested cell migration using a wound healing assay. We found that neither OE nor KD cells were distinct from the paired control cells **(** **Figures 2G, H** **)**. These results suggest that HPDL had a positive effect on the progression of PDAC *in vitro* but did not influence migration.

### HPDL Localizes to the Mitochondrial Intermembrane Space

**Figure 3:**
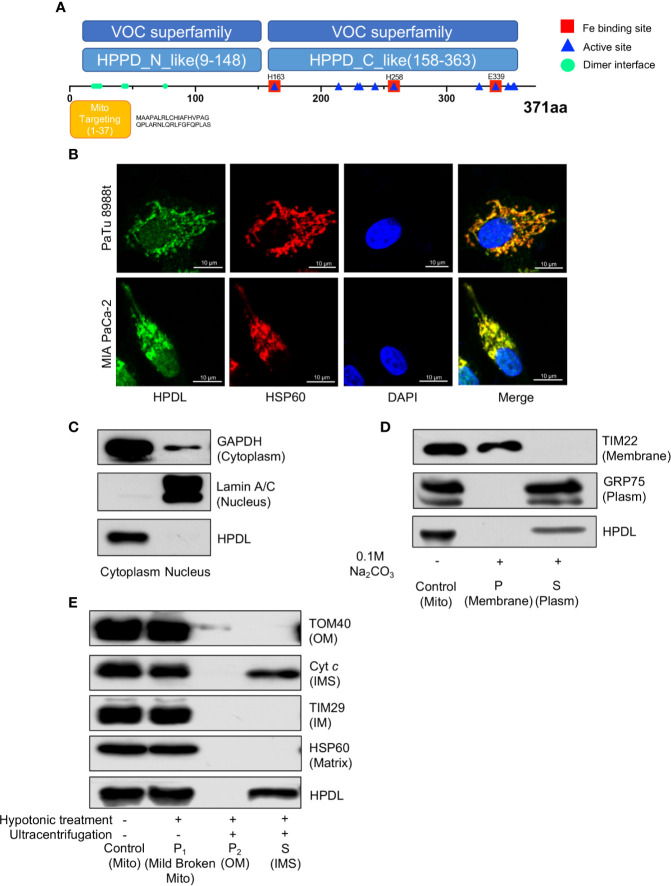
HPDL localizes to mitochondrial intermembrane space. **(A)** Schematic diagram of HPDL protein domain structure. HPPD_N_like and HPPD_C_like, C-terminal or N-terminal domain of 4-hydroxyphenylpyruvate dioxygenase (HppD) and hydroxymandelate synthase (HmaS). **(B)** Immunofluorescence colocalization analysis of HPDL in PaTu 8988t and MIA PaCa-2. Cells were immunolabeled with HPDL (Alexa flour 488, green) and HSP60 (Alex flour 594, red), and nuclear was stained with DAPI (blue). **(C)** Subcellular fractionation analysis of HPDL in MIA PaCa-2. Cytosolic and nuclear protein was extracted and immunoblotted with antibodies as indicated. **(D)** Carbonate extraction analysis of HPDL in MIA PaCa-2. Isolated mitochondria were subjected to 0.1 M Na_2_CO_3_. Mitochondrial proteins in membrane and plasm was separated with ultracentrifugation. Proteins were separated with SDS-PAGE and immunoblotted with antibodies as indicated. Mito, isolated crude mitochondria. P, pellet. S, supernatant. **(E)** Submitochondrial fractionation analysis of HPDL in MIA PaCa-2. Isolated mitochondria were subjected to hypotonic buffer (10 mM HEPES/KOH, pH 7.40) to break the integrity of mitochondrial outer membrane. Mild broken mitochondria were separated with low-speed centrifugation. Proteins in mitochondrial outer membrane and intermembrane space were subsequently separated with ultracentrifugation. Proteins were separated with SDS-PAGE and immunoblotted with antibodies as indicated. Mito, isolated crude mitochondria. P_1_, pellet in first centrifugation. P_2_, pellet in second centrifugation. OM, outer membrane. S, supernatant in second centrifugation. IMS, intermembrane space.

To probe the molecular function and biological process of HPDL, we performed domain and subcellular localization analyses. A domain analysis by the Conserved Domain Database (CDD) (41) is shown in a schematic diagram **(** **Figure 3A** **)**. HPDL was predicted to contain two 4-hydroxyphenylpyruvate dioxygenase (HppD) like domains that belongs to the vicinal oxygen chelate (VOC) superfamily, which consists of structurally related metalloproteins with distinct functions (42). We further searched out active sites in HPDL and found that HPDL contained an iron biding site like other members in VOC superfamily. Moreover, there are 37 amino acid residues in the N-terminal targeting to mitochondria as predicted by the MitoProt server (39), which further strengthens our expectation that HPDL is a mitochondrial protein.

We then performed an immunofluorescence colocalization analysis in the pancreatic tumor cell lines PaTu 8988t and MIA PaCa-2. In the colocalization analysis, HSP60 was used as a mitochondrial tracker and had a strong overlap with HPDL **(** **Figure 3B** **)**. In the fractionation of cellular components, we found HPDL colocalized with cytoplasmic protein GAPDH **(** **Figure 3C** **)**. In addition, submitochondrial localization of HPDL was performed using carbonate extraction analysis, a method used to separate hydrophobic membrane proteins and soluble proteins in mitochondria (43). The carbonate extraction analysis showed HPDL localized in the aqueous compartments, including the intermembrane space (IMS) and matrix **(** **Figure 3D** **)**. To discriminate between IMS and matrix proteins, we performed hypotonic treatment to mitochondria to break the outer membrane and release the IMS proteins, following ultracentrifugation to yield IMS proteins. Finally, HPDL was found to be localized to the intermembrane space using this more delicate submitochondrial fractionation analysis **(** **Figure 3E** **)**.

### HPDL Promotes Mitochondrial Respiration and Adenosine Triphosphate Generation

**Figure 4:**
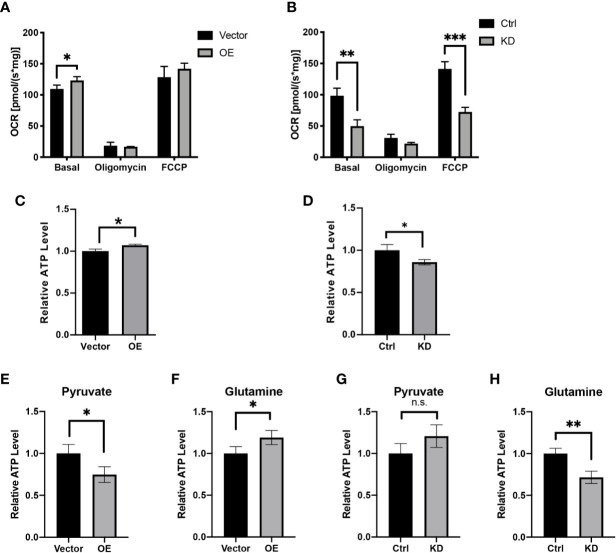
HPDL promotes mitochondrial respiration and ATP generation. **(A, B)** Oxygen consumption rate of HPDL OE **(A)** and KD **(B)** cells with theirs paired control cells. Basal, basal respiration with endogenous substrates solely. Oligomycin, respiration in the presence of ATP synthase inhibitor oligomycin (2.5 μM). FCCP, respiration in the presence of uncoupler FCCP (5 μM). **(C, D)** Relative ATP level of HPDL OE **(C)** and KD **(D)** cells with theirs paired control cells. **(E–H)** Relative ATP level of HPDL OE **(E, F)** and KD **(G, H)** cells with theirs paired control cells in different culture conditions. Cells were cultured in medium supplemented with 5 mM pyruvate only **(E, G)** or 4 mM glutamine only **(F, H)** for 12 h and subsequently the cellular ATP was measured. (*P < 0.05. **P < 0.01. ***P < 0.001. n.s., no significance.)

Having established solid confirmation of HPDL’s localization in mitochondria, we next focused on understanding its possible effects on mitochondria. We first performed an oxygen consumption rate (OCR) assay with HPDL OE and KD cells **(** **Figures 4A, B** **)**. As an inhibitor of ATP synthase, oligomycin was added to disrupt OXPHOS and then the non-phosphorylation proton flux of cells can be measured. In addition, FCCP was used as an OXPHOS uncoupler to transport protons freely through mitochondrial membrane and then the maximum uncoupled respiration can be measured. As a result, overexpression of HPDL enhanced basal respiration and knockdown of HPDL diminished basal respiration and maximum uncoupled respiration. Consistent with the OCR results, cellular ATP was also increased in OE cells and decreased in KD cells **(** **Figures 4C, D** **)**. Considering the ATP generated in glycolysis, we further deprived glucose in the culture medium and added pyruvate or glutamine instead. Unlike glucose, pyruvate and glutamine will directly enter the TCA cycle in mitochondria and generate ATP. As a result, a consistent pattern was observed in the glutamine group **(** **Figures 4F, H** **)**, but not in the pyruvate group **(** **Figures 4E, G** **)**, indicating that glutamine-dependent ATP generation was promoted by HPDL.

To further determine the effect of HPDL on mitochondrial respiration, we also performed blue native PAGE (BNG) analysis with digitonin-permeabilized cells to determine the steady level of mitochondrial OXPHOS complexes and supercomplexes. In BNG, HPDL only had a modest effect on respiratory complexes and supercomplex assembly **(** **Supplementary Figures 1A, B** **)**, suggesting that the promotive effects of HPDL on OCR are separate from OXPHOS complexes. Moreover, the mitochondrial DNA (mtDNA) copy number was shown to be unchanged in the results of quantitative PCR **(** **Supplementary Figures 1C, D** **)**. Additionally, a cellular citrate assay, a quantitative marker for the cellular mitochondria number, was performed to exclude the possible change in mitochondrial number **(** **Supplementary Figures 1E, F** **)**. Confocal and transmission electron microscopy used to investigate mitochondrial morphology found that neither individual mitochondria nor mitochondrial networks showed any significant differences **(** **Supplementary Figure 2** **)**. Taken together, these results showed that HPDL promotes mitochondrial respiration and ATP generation in PDAC but does not affect the steady level of OXPHOS complexes. Furthermore, HPDL does not affect mtDNA or mitochondrial morphology.

### HPDL Reprograms Metabolic Profiles in PDAC Cells

**Figure 5:**
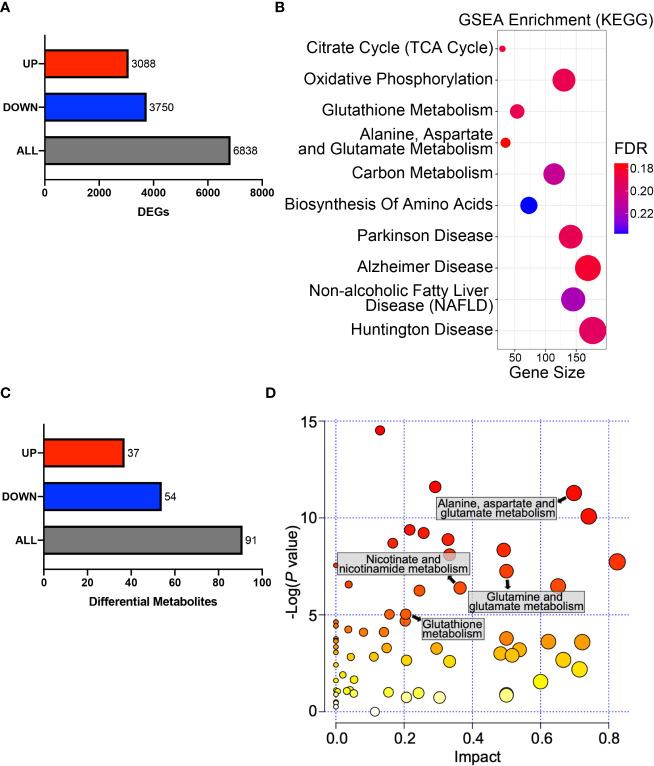
HPDL reprograms metabolic profiles in PDAC cells. **(A)** DEGs in the gene expression profiling results of HPDL OE and control cells. Threshold was set as follows: p-adj<0.05, |log_2_(OE/Vector) |>0. **(B)** GSEA in the gene expression profiling results of HPDL OE and control cells. Gene size refers to the count of genes enriched in certain pathways. FDR, false discovery rate. The GSEA was performed based on KEGG database (https://www.genome.jp/kegg/). **(C)** Differentially metabolites in metabolomic profiling results of HPDL OE and control cells. Threshold was set as follows: p<0.05, OE/Vector>1.2 or < 0.83. **(D)** MSEA in metabolomic profiling results of HPDL OE and control cells. Different metabolic pathways were plotted with p-value and impact. The MSEA was performed based on KEGG database (https://www.genome.jp/kegg/).

To obtain a general understanding of HPDL, we performed gene expression profiling on HPDL OE cells. Gene expression profiling revealed 6838 differentially expressed genes (DEGs), of which 3,088 were upregulated and 3,750 were downregulated **(** **Figure 5A** **)**. To identify and interpret these DEGs, gene set enrichment analysis (GSEA) was performed to enrich common pathways of DEGs **(** **Figure 5B** **)**. As expected, we found that HPDL was strongly associated with genes associated with in mitochondria, including the TCA cycle, OXPHOS, and a series of metabolic diseases with mitochondrial dysfunction, such as Parkinson’s disease, Alzheimer’s disease, nonalcoholic fatty liver disease(NAFLD), and Huntington’s disease (44–47). Moreover, genes involved in amino acid metabolism and carbon metabolism were enriched, indicating a possible change in the cellular metabolism of HPDL OE cells. Apart from GSEA, we also found 23 DEGs in the pancreatic cancer pathway of KEGG, including 17 oncogenes and 5 tumor suppressor genes, demonstrating the promotive effect of HPDL on PDAC **(** **Supplementary Figure 3** **)**.

Pancreatic cells develop metabolic reprograming in the malignant progression of PDAC (9). To further determine the metabolic effect of HPDL, we performed metabolomic profiling on HPDL OE cells and found 91 differential metabolites **(** **Figure 5C** **)**. Metabolite set enrichment analysis (MSEA) is a method designed to analyze metabolomic data (48). In line with the results of GSEA, we also found an association between HPDL and metabolites in glutamate and glutamine metabolism in MSEA **(** **Figures 5B, D** **)**. It has been reported that PDAC cells increase glutamine utilization in ATP generation (49). Together with our data on ATP generation **(** **Figures 4F, H** **)**, it is likely that HPDL is associated with glutamine utilization in mitochondria, however, we found that glutamine and glutamate shuttle machineries including SLC1A5_variant (23) and SLC25A12/13 (50), respectively, were not interact with HPDL (**Supplementary Data Sheet 2**). This result indicates that HPDL role in glutamine metabolism is independent of classic mitochondrial glutamine and glutamate shuttle machineries.

We also found differential metabolites in glutathione metabolism and nicotinate metabolism. Together with DEGs in glutathione metabolism, these data strongly suggest that HPDL may be associated with redox balance **(** **Figures 5B, D** **)**. Collectively, our results support HPDL’s role in reprograming metabolic profiles in PDAC, especially glutamate and glutamine metabolism as well as the metabolism of cellular antioxidants in PDAC.

### HPDL Regulates Redox Balance *via* a Glutamine-Dependent Antioxidative Pathway

**Figure 6:**
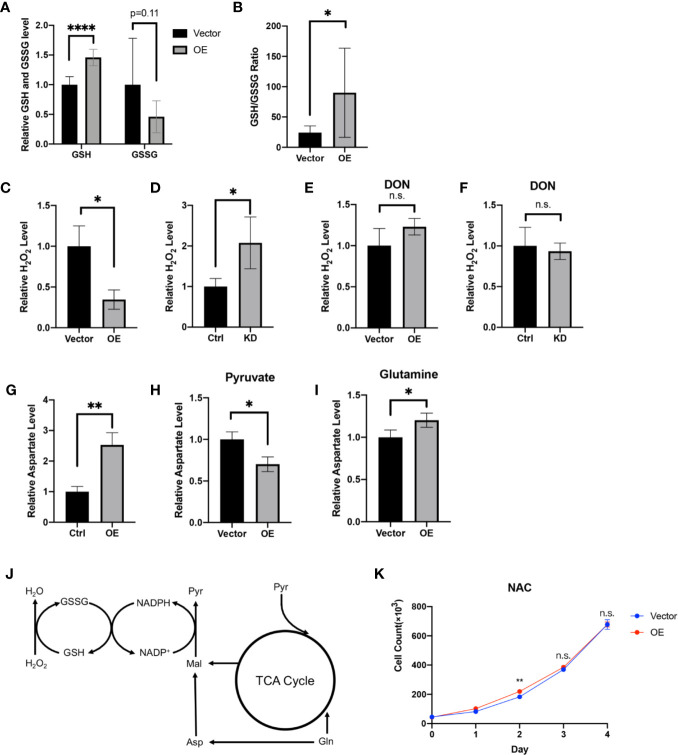
HPDL regulates redox balance *via* a glutamine-dependent antioxidative pathway. **(A, B)** Relative GSH, GSSG level **(A)** and GSH/GSSG ratio **(B)** in metabolome results of HPDL OE and control cells. **(C, D)** Relative H_2_O_2_ level of HPDL OE **(C)** and KD **(D)** cells with theirs paired control cells. **(E, F)** Relative H_2_O_2_ level of HPDL OE **(E)** and KD **(F)** cells with theirs paired control cells in the presence of glutamine antagonist DON (5 mM). **(G)** Relative aspartate level of HPDL OE and control cells. **(H, I)** Relative aspartate level of HPDL OE and control cells in different culture conditions. Cells were cultured in medium supplemented with 5 mM pyruvate **(H)** or 4 mM glutamine **(I)** as the only carbon source for 12 h and subsequently the cellular aspartate was measured. **(J)** Schematic metabolic pathway of cellular antioxidative process. This diagram was prepared based on a previous work of Son (18). Pyr, pyruvate. Gln, Glutamine. Asp, aspartate. Mal, malate. **(K)** Cell proliferation of HPDL OE and control cells with NAC treatment. Cells were cultured in medium supplemented with 5 mM NAC. (*P < 0.05. **P < 0.01. ****P < 0.0001. n.s., no significance.)

GSH is an antioxidant and acts as a scavenger of ROS in cells. In the progression of tumor cells, GSH is positively associated with tumor proliferation (51). Looking to our metabolomic data generated from HPDL OE and vector cells, we found that GSH and its oxidized form glutathione disulfide (GSSG) underwent a dramatic change **(** **Figures 6A, B** **)**. Increased GSH level and GSH/GSSG ratio in HPDL OE cells may suggest that HPDL plays a positive role in redox balance. We also found that H_2_O_2_ levels decreased in OE cells and increased in KD cells **(** **Figures 6C, D** **)**. Glutamine is used as a material for glutathione synthesis (52). In the presence of the glutamine antagonist 6-diazo-5-oxo-l-norleucine (DON), the H_2_O_2_ level remained unchanged in both HPDL OE and KD cells **(** **Figures 6E, F** **)**. In short, our data showed that HPDL is involved in the maintenance of redox balance, which is dependent on glutamine-derived GSH. In the biosynthesis of GSH, nicotinamide adenine dinucleotide phosphate (NADP^+^) and reduced NADP^+^ (NADPH) act as cofactors for glutathione reductase (GR) (53). Glutamine-derived aspartate is essential for generating NAPDH, and aspartate partially maintains redox balance (18). Our data showed that aspartate levels increased in OE cells **(** **Figure 6E** **)**. We further cultured OE cells in medium supplemented with either pyruvate or glutamine as the only carbon source and tested their aspartate levels. We found that the aspartate levels of cells using glutamine increased in a similar manner to cells cultured in complete medium, suggesting that the increase in aspartate was derived from glutamine **(** **Figures 6H, I** **)**.

In summary, we drew a schematic overview of this glutamine-dependent antioxidative pathway. Glutamine-derived aspartate is converted to malate and subsequently convert to pyruvate following generation of NADPH. NADPH will then help to reduce GSSG to GSH, which results in the removal of H_2_O_2_ **(** **Figure 6J** **)**. In addition, we cultured OE cells in the presence of antioxidant N-acetylcysteine (NAC). NAC is a precursor of cysteine and helps in the biosynthesis of GSH (54). Intriguingly, HPDL only had a modest effect on cell proliferation in the presence of NAC **(** **Figure 6K** **)**. Overall, our results found that HPDL regulates redox balance *via* a glutamine-dependent antioxidative pathway, which may be the key event in explaining the positive effect on tumor proliferation of HPDL.

## Discussion

Over the past decade, numerous studies have reported that mitochondria are involved in tumor progression. The Warburg effect drives tumor cells to oxidize glucose *via* glycolysis but not to fuel ETC (13). However there is accumulating evidence that mitochondria still affect tumor cells pleiotropically including anabolic process, redox balance, cell signaling, and cell death (15). As hubs for metabolic processes, mitochondria also play a significant role in PDAC (55). Additionally, with regard to PDAC’s association with mitochondrial dysfunction, extensive investigation of mitochondria-targeting chemotherapeutic drugs has been performed (14). In this study, we provide a comprehensive view of HPDL, a mitochondrial protein, which has not previously been fully studied. According to bioinformatic analysis and IHC analysis, we found that HPDL was upregulated in PDAC, and high expression of HPDL was associated with poor prognosis. We also generated an overexpression model and knockdown model of HPDL on PDAC cell lines and found that HPDL promoted PDAC proliferation but not migration. To some extent, HPDL has an oncogenic effect on PDAC. This oncogenic effect was further confirmed using gene expression profiling, where the typical PDAC oncogene *KRAS* was upregulated in OE cells and tumor suppressor genes *TP53* and *CDKN2A* were downregulated in OE cells **(** **Supplementary Figure 3** **)**.

As a mitochondrial protein, HPDL also participates in the mitochondrial bioenergetic process. Our data showed that HPDL promoted mitochondrial respiration and ATP generation, suggesting a positive effect of HPDL on mitochondria. We made a preliminary attempt to explain this positive effect of HPDL on PDAC. We tested a series of possible changes in mitochondria, including the steady level of OXPHOS complexes, OXPHOS supercomplex assembly, mtDNA copy number, mitochondrial content, and mitochondrial morphology **(** **Supplementary Figures 1** and **2** **)**, but our results did not support a specific explanation. We next focused on mitochondrial metabolism. Glutamine-dependent ATP generation was observed in our data, indicating a possible effect of HPDL on metabolic reprogramming.

Tumor cells develop a series of metabolic reprogramming mechanisms to meet the anabolic and bioenergetic demands of cells in a continually changing microenvironment (4). PDAC also develops a series of metabolic reprogramming mechanisms, in which *KRAS* is considered a precursor oncogene (9). The primary effect of *KRAS* in PDAC is promoting glucose uptake and the anabolic glucose pathway (56). In the process of glutaminolysis, by which the glutamine fluxes into metabolites in the tricarboxylic acid (TCA) cycle, *KRAS* redirects glutamine flux through aspartate transaminases (GOT1) other than glutamate dehydrogenase (GLUD1). At the same time, *KRAS* increases the NADPH/NADP^+^ ratio, which maintains the redox balance in PDAC (18). Although the mechanism by which HPDL affects mitochondrial respiration remains unknown, we still found evidence of its effect on metabolic reprogramming. In this study, we observed that glutamine-dependent ATP generation was enhanced by HPDL **(** **Figures 4F, H** **)**, which could suggest that glutamine is entering the TCA cycle and subsequently contributing to mitochondrial respiration. Moreover, both gene expression profiling and metabolomic profiling of HPDL OE cells strongly implicated the metabolic reprogramming of glutamine metabolism **(** **Figures 5B, D** **)**. As a carbon and nitrogen source for bioenergetic and biosynthesis process, glutamine plays a key role in cancer growth (18). GOT1 is a universally expressed enzyme that transfers nitrogen from glutamine-derived glutamate to oxaloacetate to produce aspartate and α-ketoglutarate (13). In our data, we observed a glutamine-dependent aspartate increase in HPDL OE cells **(** **Figure 6I** **)**, further suggesting that HPDL reprograms glutamine metabolism in PDAC. While HPDL is not a component of classic glutamine/glutamate transporter in mitochondria (**Supplementary Data Sheet 2**), this protein may be involved in downstream glutaminolysis pathway; however, more works are required to validate this hypothesis.

According to our immunofluorescence colocalization analysis in PDAC cells, HPDL localizes to mitochondria. This result is in line with a previous study performed on mouse neuroblastoma cell line N2a (25). Meanwhile, we also found HPDL localized in the nuclear area in the immunofluorescence analysis, this was subsequently considered to be a result of fluorescence contamination, as western blotting analysis of nuclear protein showed no HPDL existence **(** **Figure 3C** **)**. Through a series of submitochondrial fractionation, we have further demonstrated that HPDL is localized to the mitochondrial intermembrane space, where considerable amounts of ROS from respiratory complex III are generated (57). The redox balance in the IMS is tightly regulated (58). The IMS-localized proteins superoxide dismutase (Sod1) and cytochrome *c* peroxidase (Ccp1), together with glutathione redox buffer make up the IMS antioxidant system (58). Like other antioxidative enzymes in IMS, HPDL also contains an iron binding site, which may be the active site for its antioxidative capacity **(** **Figure 3A** **)**.

In our gene expression profiling and metabolomic profiling data, we observed that HPDL OE cells experienced a metabolic alteration of glutathione **(** **Figures 5B, D** **)**. Further experiments demonstrated that HPDL was associated with better regulation and control of redox balance in PDAC, which was in a glutamine-dependent pattern. The same pattern was observed by Son et al. (18), whose work on PDAC cells showed that glutamine deprivation resulted in ROS production. Moreover, Son et al. have shown that glutamine-derived NADPH that helps redox balance is critical for PDAC growth (18). Therefore, we propose this positive effect on the redox balance of HPDL may help explain its proliferative effect on tumor cells.

## Conclusion

We conclude that HPDL has a promoting effect on PDAC, and this effect may be related to glutamine metabolism and redox balance. Although the specific pathway or enzymatic process in which HPDL participates remains unknown, we still provide a constructive direction for further study on HPDL.

## Data Availability Statement

Transcriptome sequencing raw data presented in this study can be found in Genome Sequence Archive (GSA) for Human with accession number HRA000414. Raw data of LC/MS analysis can be found in **Supplementary Material**.

## Ethics Statement

The studies involving human participants were reviewed and approved by Ethical Committee of the First Affiliated Hospital of Wenzhou Medical University. Written informed consent for participation was not required for this study in accordance with the national legislation and the institutional requirements.

## Author Contributions

XY performed the experiments and wrote this manuscript. XW, JLi, PC, XL, YC, and YY performed the experiments. HS, LP, GC, and XH collected the tissue samples and performed the histological diagnosis. QZ performed the bioinformatics analysis. JLy and HF designed the experiments and revised the manuscript. All authors contributed to the article and approved the submitted version.

## Funding

This project was supported by National Natural Science Foundation of China (grant number: 31670784 and 82072366), Natural Science Foundation of Zhejiang Province (grant number: Y20H160175), Research Fund of National Health Commission, Major Medical Research Program of Zhejiang Province (grant number: WKJ-ZJ-1929), and Technology and Science Project of Yuhuan City (grant number: 201741).

## Conflict of Interest

The authors declare that the research was conducted in the absence of any commercial or financial relationships that could be construed as a potential conflict of interest.

## Supplementary Material

Supplementary Figure 1
**(A, B)** Mitochondrial respiratory complexes analysis of HPDL OE **(A)** and KD **(B)** cells with theirs paired control cells. Cells were permeabilized with 2.5% digitonin and protein was separated by 3%-11% blue native PAGE. Mitochondrial respiratory complexes were immunoblotted with anti-Grim19 (CI), SDHA (CII), UQCRC2 (CIII), MT-CO1 (CIV) and ATP5A (CV). SCs, super complexes. CI-CV, mitochondrial respiratory complexes I-V. TOM70 was used as a loading control. Quantitative results were shown in the right panel. **(C, D)** Relative mtDNA copy number of HPDL OE **(C)** and KD **(D)** cells with theirs paired control cells. DNA was extracted from cell and mtDNA was determined by quantitative PCR. **(E, F)** Relative citrate synthase activity of HPDL OE **(E)** and KD **(F)** cells with theirs paired control cells. Cell were disrupted by snap-freeze and citrate synthase activity was determined with 0.3 mM acetyl-CoA, 0.5 mM OAA, and 0.1 mM DTNB. (n.s., no significance.)

Supplementary Figure 2
**(A, B)** Mitochondrial morphology analysis of HPDL OE **(A)** and KD **(B)** cells with theirs paired control cells by immunofluorescence staining. Mitochondria were stained with green fluorescence (HSP60) using immunofluorescence staining. Median branch length of individual mitochondria and mitochondrial networks size were measured, and quantitative results was shown in lower panel. **(C, D)** Mitochondrial morphology analysis of HPDL OE **(C)** and KD **(D)** cells with theirs paired control cells by transmission electron microscopy. Mitochondria were shown in different zoom level. (n.s., no significance.)

Supplementary Figure 3 Heatmap of oncogenes and tumor suppressor genes in HPDL OE cells. The heatmap was generated based on the data of gene expression profiling.

Supplementary Table 1 Primary and secondary antibodies.

Supplementary Data Sheet 1 Quasi-targeted metabolomic analysis of HPDL OE cells.

Supplementary Data Sheet 2 LC-MS analysis of immunoprecipitation of FLAG-tagged HPDL proteins.
