# Supplementary figures and images for: 4-Hydroxyphenylpyruvate Dioxygenase-Like Protein Promotes Pancreatic Cancer Cell Progression and Is Associated With Glutamine-Mediated Redox Balance
Source: Front Oncol. 2021 Jan 18;10:617190. doi: 10.3389/fonc.2020.617190 (PMC7848781; doi:10.3389/fonc.2020.617190)

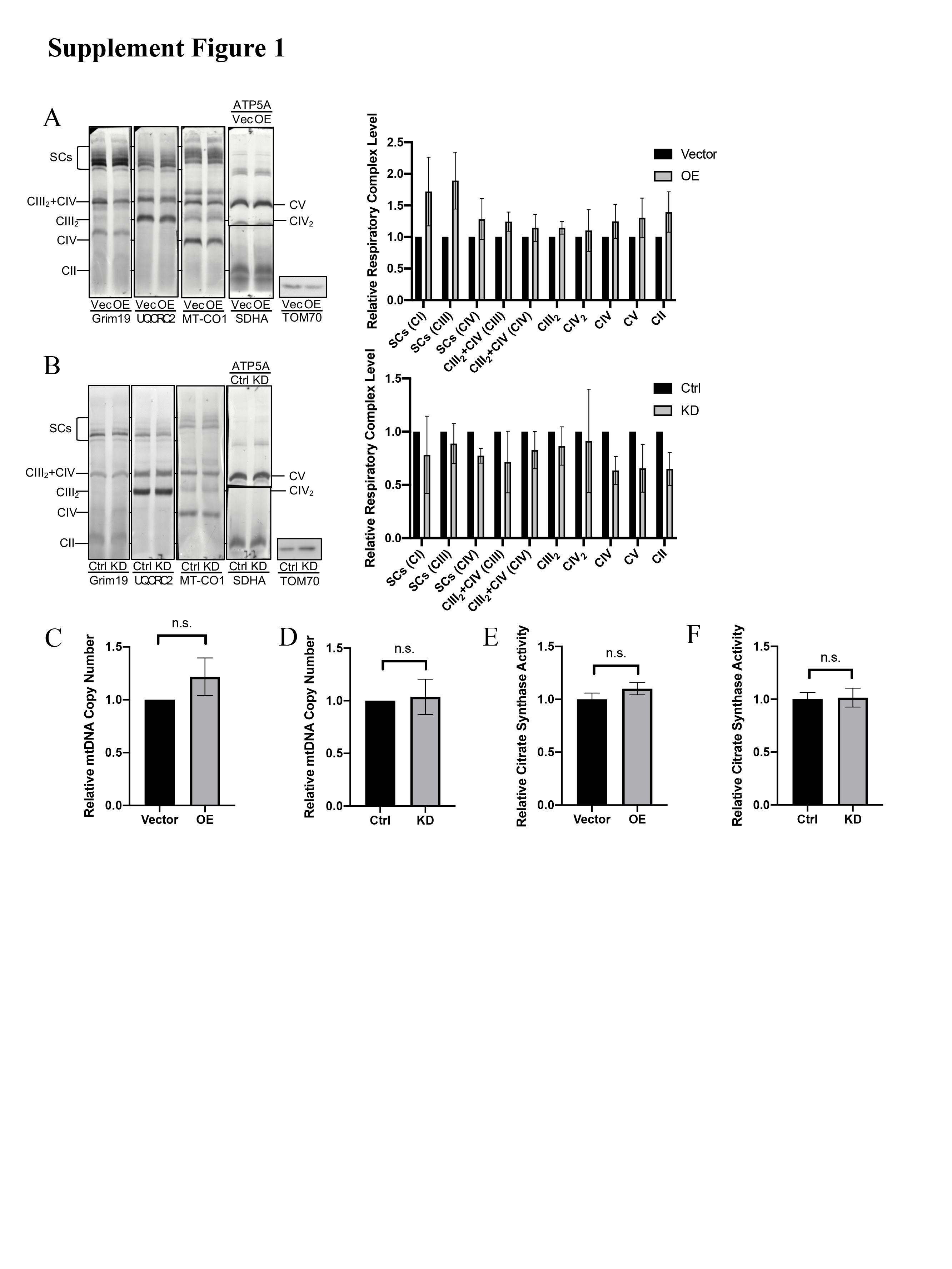

Supplement: Supplementary Figure 1 — (A, B) Mitochondrial respiratory complexes analysis of HPDL OE (A) and KD (B) cells with theirs paired control cells. Cells were permeabilized with 2.5% digitonin and protein was separated by 3%-11% blue native PAGE. Mitochondrial respiratory complexes were immunoblotted with anti-Grim19 (CI), SDHA (CII), UQCRC2 (CIII), MT-CO1 (CIV) and ATP5A (CV). SCs, super complexes. CI-CV, mitochondrial respiratory complexes I-V. TOM70 was used as a loading control. Quantitative results were shown in the right panel. (C, D) Relative mtDNA copy number of HPDL OE (C) and KD (D) cells with theirs paired control cells. DNA was extracted from cell and mtDNA was determined by quantitative PCR. (E, F) Relative citrate synthase activity of HPDL OE (E) and KD (F) cells with theirs paired control cells. Cell were disrupted by snap-freeze and citrate synthase activity was determined with 0.3 mM acetyl-CoA, 0.5 mM OAA, and 0.1 mM DTNB. (n.s., no significance.) [file Image_1.jpeg]

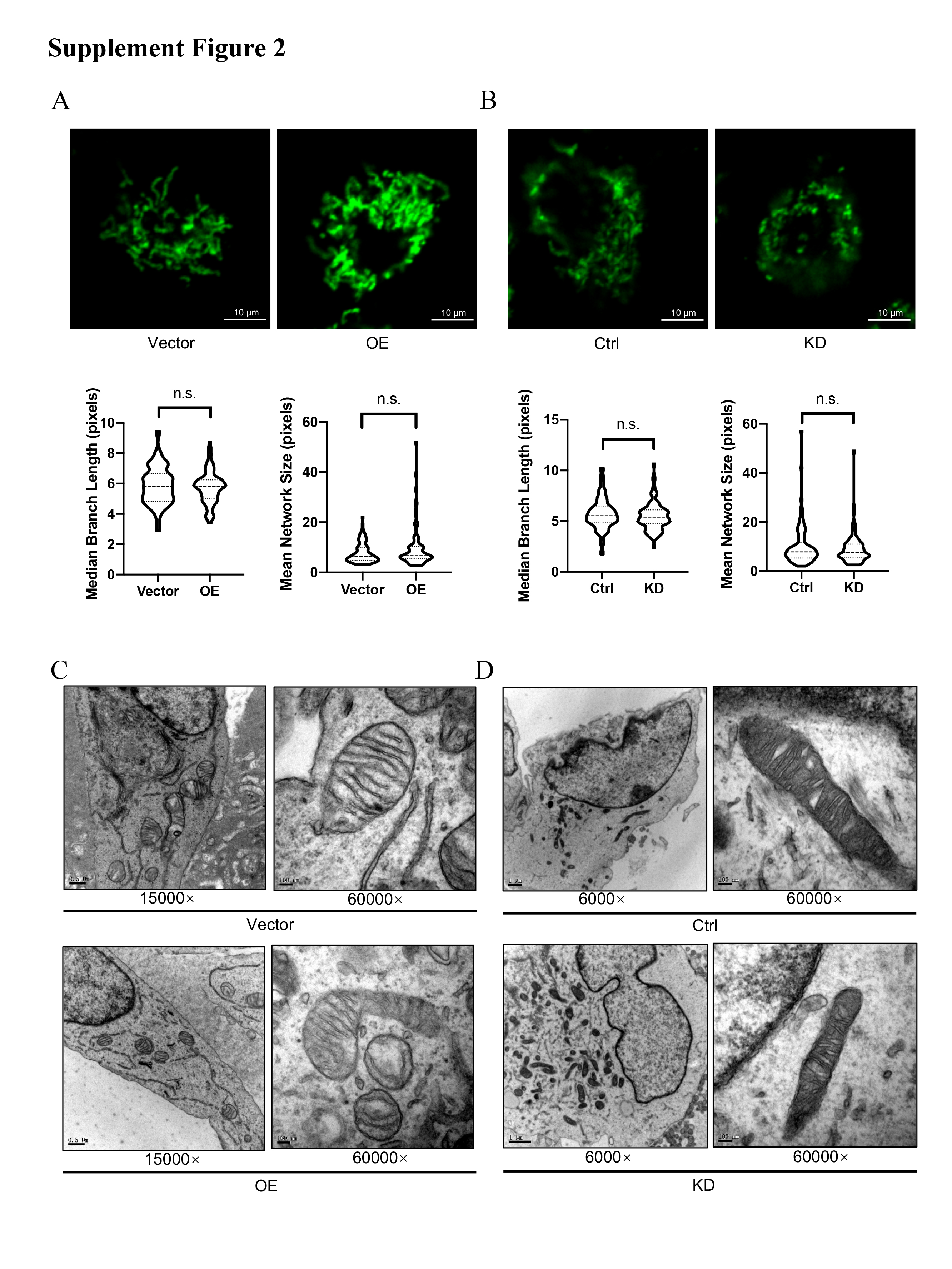

Supplement: Supplementary Figure 2 — (A, B) Mitochondrial morphology analysis of HPDL OE (A) and KD (B) cells with theirs paired control cells by immunofluorescence staining. Mitochondria were stained with green fluorescence (HSP60) using immunofluorescence staining. Median branch length of individual mitochondria and mitochondrial networks size were measured, and quantitative results was shown in lower panel. (C, D) Mitochondrial morphology analysis of HPDL OE (C) and KD (D) cells with theirs paired control cells by transmission electron microscopy. Mitochondria were shown in different zoom level. (n.s., no significance.) [file Image_2.jpeg]

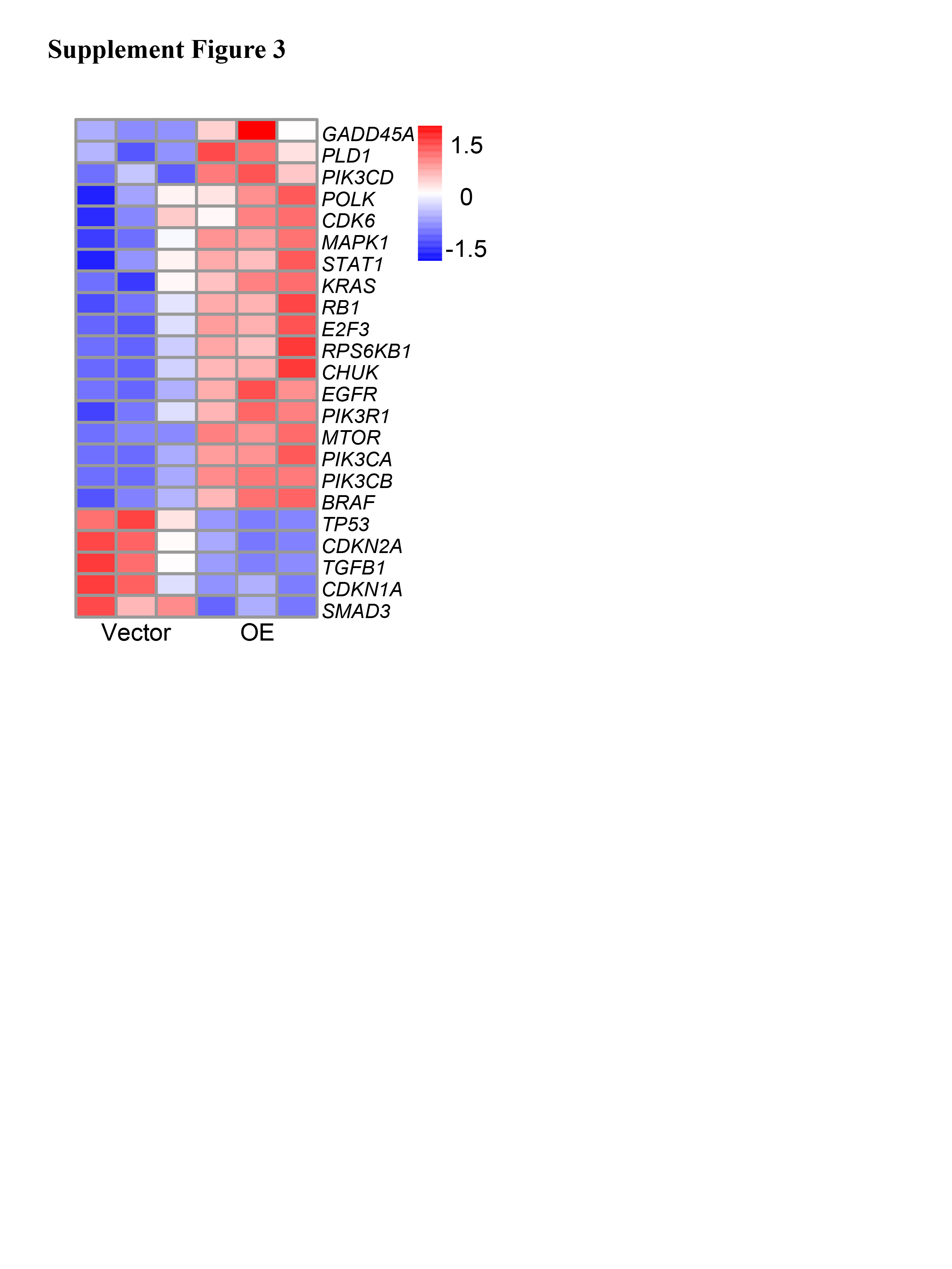

Supplement: Supplementary Figure 3 — Heatmap of oncogenes and tumor suppressor genes in HPDL OE cells. The heatmap was generated based on the data of gene expression profiling. [file Image_3.jpeg]
